# Interactions among Odorants, Phenolic Compounds, and Oral Components and Their Effects on Wine Aroma Volatility

**DOI:** 10.3390/molecules25071701

**Published:** 2020-04-08

**Authors:** María Perez-Jiménez, Adelaida Esteban-Fernández, Carolina Muñoz-González, María Angeles Pozo-Bayón

**Affiliations:** Instituto de Investigación en Ciencias de la Alimentación (CIAL) CSIC-UAM.C./Nicolás Cabrera, 9, 28049 Madrid, Spain; maria.perez@csic.es (M.P.-J.); c.munoz@csic.es (C.M.-G.)

**Keywords:** wine, aroma volatility, aroma-matrix interactions, polyphenols, oral physiology, saliva, buccal cells

## Abstract

To determine the impact of oral physiology on the volatility of typical wine aroma compounds, mixtures of a synthetic wine with oral components (centrifuged human saliva (HS), artificial saliva with mucin (AS), and buccal epithelial cells (BC)) were prepared. Each wine type was independently spiked with four relevant wine odorants (guaiacol, β-phenyl ethanol, ethyl hexanoate, and β-ionone). Additionally, the impact of four types of phenolic compounds (gallic acid, catechin, grape seed extract, and a red wine extract) on aroma volatility in the HS, AS, and BC wines was also assessed. Static headspace was measured at equilibrium by solid phase microextraction–GC/MS analysis. Results showed a significant impact of oral components on the volatility of the four tested odorants. Independently of the type of aroma compound, aroma volatility was in general, higher in wines with BC. Moreover, while guaiacol and ethyl hexanoate volatility was significantly lower in wines with HS compared to wines with AS, β-ionone showed the opposite behavior, which might be related to metabolism and retention of mucin, respectively. Phenolic compounds also showed a different effect on aroma volatility depending on the type of compound and wine. Gallic acid had little effect on polar compounds but it enhanced the volatility of the most hydrophobic ones (ethyl hexanoate and β-ionone). In general, flavonoid type polyphenols significantly reduced the volatility of both polar (guaiacol and β-phenyl ethanol) and hydrophobic compounds (β-ionone in HS and BC wines), but through different mechanisms (e.g., π–π interactions and hydrophobic binding for polar and apolar odorants respectively). On the contrary, flavonoids enhanced the volatility of ethyl hexanoate, which might be due to the inhibition exerted on some salivary enzymes (e.g., carboxyl esterase) involved in the metabolism of this odorant molecule.

## 1. Introduction

To be perceived, odorant volatile compounds needs to be released from the wine matrix to the air flows. The release of volatiles in the airflow depends on the presence and concentration of volatiles in the vapor phase and on all the factors influencing aroma partition between the vapor and the liquid (wine) [1]. The physicochemical nature of the aroma compound (volatility, hydrophobicity, etc.) and the wine chemical composition (polyphenols, ethanol, polysaccharides, etc.) can determine the binding of aroma compounds to different wine matrix components [2].

Previous studies on wine-aroma interactions have been conducted considering the impact of single wine components (e.g., polyphenols, polysaccharides) [3,4,5,6,7], or several wine matrix components, by using more complex synthetic wines [8,9] or reconstituted wines [10,11]. All of them have provided valuable information on the outstanding role of some wine matrix components on aroma volatility highlighting the necessity to consider aroma–wine matrix interactions, and not only the wine aroma profile when trying to explain wine aroma perception [12].

However, and differently to what happens when a wine is smelt and its odor is perceived (orthonasal pathway), during eating or drinking, aroma compounds are released in the mouth and/or in the throat, and they travel pushed by the expiration flows to the olfactory receptors. Once in the mouth, wine components (volatile and non-volatile) can also interact with oral fluids (saliva) and structures (oral mucosa), which might also affect aroma volatility [13].

Recent in-vivo studies have shown differences in individual patterns of in-mouth aroma release which might be associated to differences in oral physiology [14]. Salivary flow and composition have also been related to differences on in-mouth [15] and in-nose [16] wine aroma release. Unfortunately, the molecular mechanisms behind these interactions remain poorly understood.

The scarce studies trying to elucidate the nature of these interactions have been focused on the impact of saliva on wine aroma release by using different methodological approaches (static vs. dynamic headspace), wine matrix compositions (synthetic vs. real wines), tested odorants (mixtures of aroma compounds vs. original wine volatile profile), and salivary composition/treatment (e.g., saliva centrifuged/not centrifuged) [17,18,19]. This makes it difficult to extract conclusions on the nature and significance of aroma–saliva–wine matrix interactions

Additionally, recent research using aromatized solutions showed that besides the well-known dilution or salting out effects [20], saliva can act on aroma compounds at very different levels, binding different types of odorants (hydrogen, hydrophobic interactions) [21,22] and/or metabolizing them [23,24,25,26,27,28], which also might give rise to new odorant metabolites [25,27,28]. Whether these effects can be relevant in wine, and if wine matrix components might also modulate the effect exerted by saliva on aroma compounds, are some questions that need to be addressed.

Moreover, the role of other oral physiological components on wine aroma volatility is unknown. Although it has been shown that wine odorants can be adsorbed to oral mucosa [14], it is not clear if they can be directly adsorbed onto oral epithelial cells [29] or if they can bind to polyphenols from the food matrix already adsorbed to the oral epithelial cells. Actually, saliva can increase the stickiness of polyphenols to the surface of the oral mucosa [30], which might also affect the interaction with odorant compounds. Payne et al. [31] also showed that some polyphenols can directly bind to oral epithelial cells. However, as far as the authors know, there are no previous works considering the effect of saliva and buccal epithelial cells on wine aroma volatility in the same study.

Therefore, the aim of this study was to determine the relative effect of the oral physiological components (saliva, buccal epithelial cells) on wine aroma volatility, while also considering the presence of different types of wine matrix compounds (phenolic compounds). To do so, three types of wines that consisted of one synthetic wine spiked with centrifuged human saliva (containing low molecular weight proteins), artificial saliva with mucin (a glycosylate high molecular weight protein), and buccal epithelial cells were prepared. Each wine type was independently assessed with four aroma compounds of different physicochemical characteristics (guaiacol, β-phenyl ethanol, ethyl hexanoate, and β-ionone). Additionally, the effect of four types of phenolic compounds (gallic acid, catechin, grape seed extract, and a red wine extract) on aroma volatility using wines with different types of oral components was also tested. A static headspace approach at equilibrium using Solid Phase Microextraction (SPME)-GC/MS with short sampling times was followed to determine the effect of each oral and wine matrix component on aroma volatility.

## 2. Results

### 2.1. Effect of Oral Components on Wine Aroma Volatility

In this work, the relative effect of different oral components on the volatility of typical and chemically different wine odorants was assessed in synthetic wines spiked with human saliva (HS), artificial saliva (AS), and buccal epithelial cells (BC). These wine–oral systems were intended to better represent the physiological environment in the mouth during drinking, in which odorants might differently interact with oral physiological components. Static headspace analysis, which is the best suited to reveal these interactions [20], was used for this purpose. In addition, SPME using short sampling times was selected once it its appropriateness for this type of studies was previously proven [32].

First, the relative effect of the different oral component on the headspace amount (absolute peak area) of each studied aroma compounds (guaiacol, β-phenyl ethanol, ethyl hexanoate, and β-ionone) was explored by one-way ANOVA. Results showed a significant effect of oral components on aroma volatility for all tested compounds. On the basis of the F-ratios and probability (*p*) values, the highest effect of oral components was on guaiacol (F = 19.362, *p* = 0.004), followed by β-ionone (F = 16.637, *p* = 0.012), ethyl hexanoate (F = 16.282, *p* = 0.012), and β-phenyl ethanol (F = 14.675, *p* = 0.008). 

**Figure 1:**
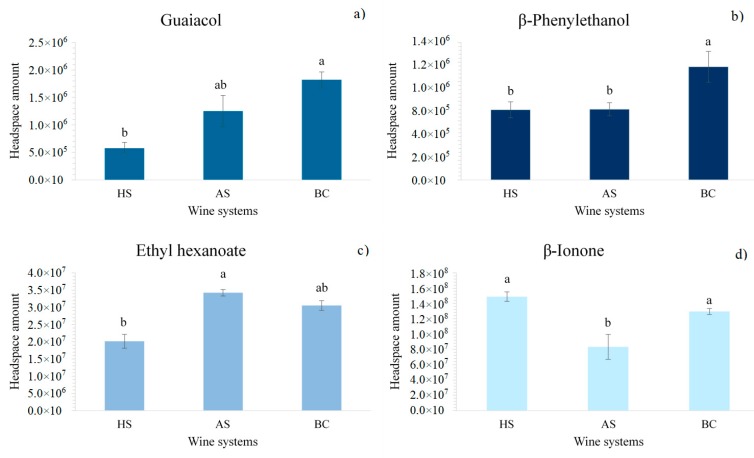
Effect of oral components (HS: human saliva, AS: artificial saliva, BC: buccal cells) added to synthetic wine on aroma volatility, (**a**) Guaiacol, (**b**) β-Phenylethano, (**c**) Ethyl hexanoate and (**d**) β-Ionone. Different letters on the top of the bars for each aroma compound denote significant differences among wine systems (*p* < 0.05) from Tukey test.

In order to determine the extent of the effect of each oral component on aroma volatility, a means comparison test (Tukey test) was also performed. Figure 1 shows these results. As it can be seen, oral components modified the volatility of the tested aroma compounds in a different way, depending on the oral component added to the wine, but also depending on the type of aroma compound. This makes it difficult to extract straightforward conclusions. However, some interesting findings can be highlighted from these results. 

Firstly, and as it can be seen in Figure 1, in general, wines with BC exhibited higher headspace aroma amounts compared with the other types of wine systems. The effect of BC seemed to be higher in the case of β-phenyl ethanol. This polar compound could be salted out by the addition of BC, which could modify its solubility in the wine. Actually, using a model of oral epithelial cells, it has been shown that the surface of the oral cells present both highly hydrophobic and hydrophilic domains due to the different expression of MUC1/Y-LSO mucin [33]. The expression of MUC1 results in the presence of more hydrophobic and more charged areas at the cell surface [33]. This might explain the change in wine polarity, making it more hydrophobic and provoking the salting out of more polar molecules. This could also explain the higher headspace amounts of guaiacol. However, this explanation might not be valid in the case of more hydrophobic compounds, such as β-ionone, which exhibited similar headspace amounts in the presence of BC. For this compound aroma volatility was similar in the wines with BC and HS, but significantly lower in the case of AS only containing mucin. The retention of this hydrophobic compound by mucin, as it has been described for other hydrophobic aroma compounds [21], might be the reason for these differences.

Other effects of epithelial cells that have been previously described are related to their ability to metabolize different types of volatile organic compounds from different chemical families [25,34,35]. However, under our experimental conditions, we could not prove this hypothesis. Additionally, by comparing the three wine systems, it was interesting to note the large difference between the volatility of most of the odorants (except β-phenylethanol) in wines spiked with HS and AS. Guaiacol and ethyl hexanoate showed the lowest volatility in systems with HS (Figure 1a,c). On the contrary, β-ionone showed the lowest volatility in the wines with AS (Figure 1d). This might be explained by the different composition of proteins of both types of saliva. AS only contained mucin, while HS, as it was previously centrifuged, should not contain this type of large glycosylated protein [27] but mainly low molecular weight proteins (proline rich proteins (PRP)s, α-amylase, cystatins, etc.). The interaction of aroma compounds with different types of salivary proteins has previously been shown [20,21]. These interactions might not have a direct relationship with aroma compound hydrophobicity [20], which explains the effect observed for guaiacol and ethyl hexanoate despite their different hydrophobicities. On the contrary, as previously described, a very low headspace amount of β-ionone was observed in the wine with AS compared to that with HS (Figure 1d), which as it has already been explained, might be due to the affinity of this odorant to bind large glycosylated proteins, which should be absent in the wine with HS. 

Another reason to explain the reduced headspace amount of some compounds, such as guaiacol and ethyl hexanoate in the wines spiked with HS (Figure 1a,c), could be their metabolism by salivary enzymes [23,24,27]. Although in the case of guaiacol, this hypothesis has never been proven, the hydrolysis of ethyl hexanoate by saliva esterase enzymes giving rise to the corresponding carboxylic acid has been proven [28]. This might explain the different volatility of these odorants in wines with AS (without metabolic capacity) and with HS (with metabolic capacity).

### 2.2. Effect of Phenolic Compounds on Aroma Volatility in Wines with Different Oral Components

**Figure 2:**
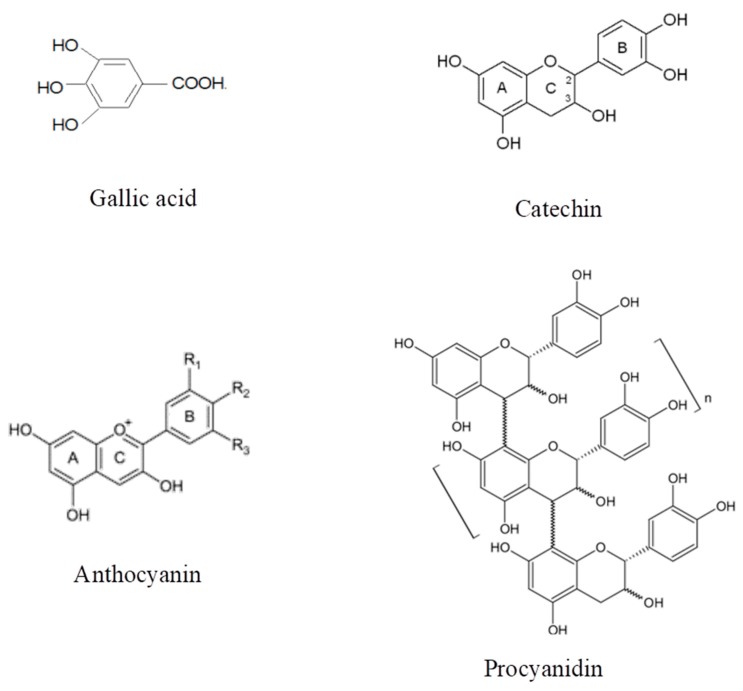
Chemical structure of the phenolic compounds employed in this study. Procyanidins and anthocyanins were the main constituents of the grape seed and red wine extracts, respectively.

In a second experiment, the role of phenolic compounds on aroma volatility was investigated using synthetic wines spiked with oral components. Phenolic compounds are quantitatively the most important group of non-volatile wine matrix components, and although their influence on wine aroma volatility has been previously investigated [4,5,6,7,8], works also considering the presence of oral components and therefore simulating a more realistic scenario of what could happen with aroma compounds in the mouth during wine tasting are quite scarce. For this experiment, the three types of synthetic wines with HS, AS, and BC were prepared and individually spiked with four different types of phenolic compounds: gallic acid (GAc), catechin (Cat), a red wine extract (RWE), and a grape seed extract (GSE). These four types of phenolic compounds exhibited different chemical structures and properties (Figure 2), and likely, a different behavior against aroma compounds and oral components. A control wine of each type (HS, AS, BC) without polyphenols was also prepared. 

**Figure 3:**
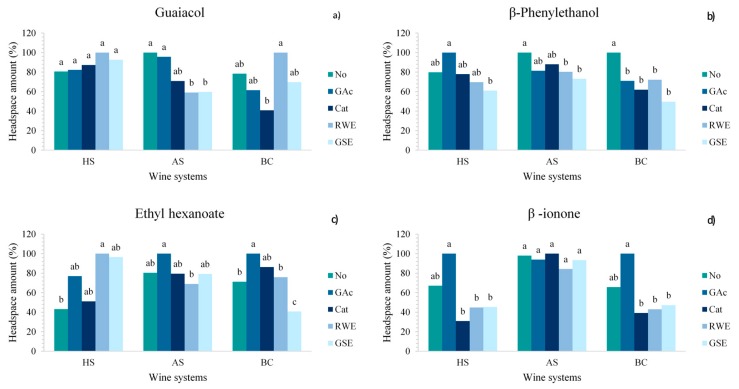
Effect of phenolic compounds (No: without polyphenol, GAc: gallic acid, Cat: catechin, RWE: red wine extract, GSE: grape seed extract) on wine aroma volatility. For each aroma compound, (**a**) Guaiacol, (**b**) β-Phenylethano, (**c**) Ethyl hexanoate and (**d**) β-Ionone, and type of wine system, different letters on the top of the bars denote significant differences among wines spiked with different polyphenols (*p* < 0.05) from Tukey test.

First, to determine the impact of the different types of phenolic compounds on the volatility of the four aroma compounds in the three synthetic wines with oral components (HS, AS, and BC), aroma volatility data (absolute peak areas) from each wine type were separately submitted to one way-ANOVA. In addition, a means comparison test (Tukey test) was also run in order to determine the individual effect of each phenolic compound. Figure 3 summarizes these results. As it can be seen, in the case of guaiacol (Figure 3a), the effect of the type of phenolic compound was only significant in the wines with AS and BC, but not with HS. In wines with AS, the control wine without phenolic compounds and the wine with gallic acid showed the highest headspace amounts of this odorant molecule, but there were not significant differences between them. This reveals that in wines with AS, the monomeric phenol gallic acid did not provoke any effect on guaiacol volatility, which is in agreement with previous studies, in which gallic acid did not show any effect on the volatility of 3-alkyl-2-methoxypyrazynes when added to a model wine at 1 g/L [36]. However, Figure 3a also reveals that wines with AS and supplemented with flavonoid type polyphenols (Cat, GSE, and RWE) exhibited the lowest headspace amount of guaiacol (above 30–40% less compared to the control). In previous works aimed to study the effect of the wine matrix on aroma volatility, interactions between some polyphenols and certain aroma compounds with an aromatic ring through a π–π staking between the galloyl ring of the polyphenol, and the aromatic ring of the odorant molecule, with stability provided by hydrogen bonding, have been described [6]. It has also been shown that this type of interaction can reduce the headspace concentration of aroma compounds in wines [4,9,10]. In more recent works using in vivo approaches, it has been suggested a possible interaction of this compound with polyphenols adsorbed to the mucosal pellicle which is the salivary film that covers the oral epithelium and is rich in MUCB and MUC7 [37] explained π–π staking. Thus, the above-proposed mechanism (π–π interactions) might be the reason for the reduction of guaiacol volatility in wines with flavonoid type polyphenols and mucin (AS). As previously described, this large protein should be absent in HS, due to the centrifugation step, explaining the lack of effect of these phenolic compounds on guaiacol volatility in wines with HS. 

In the wines with BC, a significant effect of phenolic compounds on guaiacol volatility was also observed (Figure 3a). While guaiacol volatility was more similar in the control wine (without polyphenols) than the wines with gallic acid or GSE, in the case of the wines with catechin, a strong reduction of volatility (above 50% compared to the control) was observed. The addition of RWE into the wines with BC produced a salting out effect, and therefore, an increase in guaiacol volatility above 20% compared to the control wine. The different effect of polyphenols on guaiacol volatility in wines spiked with BC compared to wines with AS could be due to the different hydrophobic properties of BC. As said before, the presence at the surface of the oral cells of highly hydrophobic and hydrophilic domains due to the expression of different types of mucin [33] might differently affect the binding of different types of phenolic compounds to BC, which in turn might also affect the interaction with guaiacol. 

In the case of β-phenyl ethanol, the effect of phenolic compounds was significant in the three types of wines (with HS, AS, and BC) (Figure 3b). In general, in all of them, the addition of these compounds produced a reduction on β-phenyl ethanol volatility compared to the control wine. Only in the case of the wines with HS did the addition of gallic acid provoke a salting out effect. Interestingly, a similar effect (an enhancement of aroma volatility) has been shown in wines with high phenolic acid content when using in-mouth aroma monitoring [38]. In general, the lowest headspace concentration of this odorant was found in the wines spiked with flavonoid type compounds, being the wines with GSE exhibiting the lowest volatility of β-phenyl ethanol. For instance, in wines with BC, the reduction of aroma volatility by adding GSE was about 50% compared to the control (Figure 3b). Considering the relatively low hydrophobicity and the presence of an aromatic ring in the β-phenyl ethanol molecule, the formation of π–π interactions with these flavonoids might also be a plausible explanation for the reduced volatility of this odorant in the presence of these types of polyphenolic compounds. 

A significant effect of polyphenols on ethyl hexanoate volatility considering the three types of wines supplemented with oral components was also found (Figure 3c). On the basis of its *log P* (hydrophobicity) value (Table 1), this compound can be considered of hydrophobic nature. Interestingly, the effect of each type of polyphenol was very different in the wine with human (HS) or artificial (AS) saliva (Figure 3c). As it can be seen (Figure 3c), in the wine with HS the lowest percentage of headspace release was found in the control wine without polyphenols. In the wines with polyphenols, and mainly in those supplemented with the two phenolic extracts (RWE and GSE), ethyl hexanoate volatility increased above 60% compared to the control wine without polyphenols. Both extracts were mainly composed of anthocyanins and procyanidins, respectively [39,40], but their effects on ethyl hexanoate volatility were very similar. Previous works have proven the existence of hydrophobic-driven, weak bimolecular aroma-phenolic compound interactions [4]. Additionally, Dufour and Sauvaitre [5] reported that at pH 3.5 (normal wine pH), malvin, the hemiacetal form of the anthocyanin 3,5 diglucoside, was primarily responsible for aroma interactions. The consequence should be a reduction on aroma volatility, even larger for a relatively high hydrophobic molecule as ethyl hexanoate. However, Figure 3c shows a higher release of this odorant in the systems with polyphenols, and mainly when they were supplemented with phenolic extracts. To explain this, one possible hypothesis can be related to the capacity of certain polyphenols to inhibit some salivary enzymatic activities (α-amylases, carboxylesterases) [41,42,43,44]. It has been demonstrated that human saliva can metabolize carboxylic esters, producing the corresponding carboxylic acids [23,28]. As previously described, the large difference in ethyl hexanoate volatility between wine with AS and HS has been associated with the hydrolysis of this compound by esterase enzymes (Figure 1c). However, this transformation should not happen if human saliva enzymes are inactivated [28]. Therefore, it is plausible to think that the higher release of this odorant in the wines with HS and phenolic compounds (Figure 3c) might due to the inhibitory effect of these types of compounds on salivary esterase, reducing the transformation of ethyl hexanoate in the corresponding volatile acid, which might happen in the HS systems without polyphenols. Furthermore, both phenolic extracts seemed to have a high inhibitory activity compared to the monomer catechin and the non-flavonoid compound gallic acid, which is in agreement with the higher inhibitory carboxylesterase capacity of flavonoid-type polyphenols [44]. This funding might also explain previous results [17] in which authors observed a higher decrease in ester release in white wines spiked with human saliva compared to red wines (with flavonoid type polyphenols) when using dynamic headspace conditions.

In the case of the wine with AS, a very similar headspace amount of ethyl hexanoate was found in the control wine compared to the wine with phenolic compounds (Figure 3c). However, a significant increase of aroma volatility produced by gallic acid and a significant decrease produced by the addition of RWE was also observed. The addition of a polar compound such as gallic acid might have modified the wine polarity, decreasing the solubility of ethyl hexanoate, which could now be better released to the head space. On the contrary, the formation of weak hydrophobic interactions between a hydrophobic odorant compound and anthocyanin type compounds [5] could reduce the volatility of ethyl hexanoate. 

Similarly to what happened for wines with AS, in the wines with BC gallic acid increased the volatility of ethyl hexanoate (Figure 3c), while the two phenolic extracts and mainly GSE decreased it, which is in agreement with the previously described hydrophobic interactions. However, the addition of catechin did not reduce ethyl hexanoate volatility, and even produced a slight increase (Figure 3c). 

Finally, phenolic compounds also affected the volatility of β-ionone in the wines with HS and BC, but not in the wines with AS (Figure 3d). The latter also exhibited the highest values of β-ionone in the headspace. This shows a higher interaction of this volatile compound with polyphenols in the wines with HS and BC compared to those with AS. In both wine types, the addition of phenolic compounds provoked practically the same effect on β-ionone volatility. For instance, the addition of gallic acid produced an enhancement in the volatility of this odorant compound compared to the control wine (above 40% increase). This salting out effect could be related to the reduction in solubility when a highly polar phenolic acid is added into the wine, as also happened for ethyl hexanoate. Since β-ionone is more hydrophobic than ethyl hexanoate, this effect is larger for the former. In presence of flavonoids, a reduction (of above 20% compared to the control) of the amount of β-ionone in the headspace was evidenced in both wine types (with HS and BC) (Figure 3d). Previous works have shown than in the presence of human saliva, it is possible the formation of complexes involving phenolic compounds and salivary proteins is able to encapsulate mainly hydrophobic compounds [18]. The formation of this type of complexes might be less favored in wines with AS, in which only one protein (mucin) is present in the medium. Moreover, the direct binding of polyphenols to oral epithelial cells has been already described [31]. This might also trigger the binding of odorant molecules, especially those of a hydrophobic nature, such as β-ionone, which also could explain the lower headspace amount of β-ionone in these type of wines with BC. 

## 3. Materials and Methods

### 3.1. Chemical and Reagents

Tartaric acid and ethanol were from Panreac Química (Barcelona, Spain). Ethyl hexanoate and β-phenyl ethanol were from Merck (Darmstadt, Germany). β-ionone was from Fluka (Buchs, Switzerland). Guaiacol from Aldrich (Steinheim, Germany), Trifluoroacetic acid (TFA), NaHCO_3_, K_2_HPO_4_.3H_2_O, KCl, gallic acid, and porcine mucin were purchased from Sigma–Aldrich (Steinheim, Germany). CaCl_2_.2H_2_O was from Montplet (Barcelona, Spain). NaOH and HCl were provided by Fisher Chemical (Loughborough, UK). Tris-buffered saline (TBS) was provided by Thermo Scientific (Waltham, MA, USA) and catechin from Sigma (St. Louis, MO, USA). NaCl and HCl were purchased from VWR (Leuven, Belgium). All the chemicals were of the highest purity available.

### 3.2. Synthetic Wines

Synthetic wines were prepared by adding 0.3 g of tartaric acid to 10 mL of absolute ethanol and 90 mL of deionized milliQ water (18.2 mΩ.cm^−1^). The pH of the solution was adjusted to 3.5 with NaOH.

### 3.3. Aroma Compounds

Four aroma compounds were used in this study (ethyl hexanoate, β-ionone, guaiacol, and β-phenyl ethanol). They were selected because they are typical wine odorants from different chemical classes having different physicochemical properties (Table 1), and likely different behaviors against oral components (saliva and buccal epithelial cells). For the aromatization, four independent stock solutions of each aroma compound (10,000 mg/L) in absolute ethanol were prepared, and from there, synthetic wines were aromatized to have different final concentrations depending on the compound (1 mg/L in the case of ethyl hexanoate, 10 mg/L for β-ionone and β-phenylethanol, and 50 mg/L in the case of guaiacol).

### 3.4. Oral Components (Human Saliva and Buccal Cells)

Stimulated human saliva (HS) samples collected from 12 volunteers (8 females and 4 males, aged 21–43 years old) were pooled and used for this study. Previously, subjects were asked to avoid eating or drinking anything different from water for at least 1 h before saliva collection. Stimulated saliva was obtained by chewing a small piece of parafilm and then spitting it out after 15 min into a sterile tube. Two saliva collections were done on the same day: one in the morning and one in the afternoon. All saliva samples were immediately stored at −20 °C and then pooled after the last collection. The pool was divided into 50 mL aliquots, which were centrifuged at 2600× *g* for 15 min at 4 °C. Supernatants were then stored at −80 °C until use for a maximal time of three weeks. The pH of human saliva samples was 7.27 and a total protein content of 980.7 ± 0.57 mg/L determined by applying the Bicinchonic Acid (BCA) assay (Pierce Thermo Scientific, IL, USA) [45].

Buccal cells (BC) were obtained from the same 12 volunteers who donated saliva. Cell collection was freshly done for each experiment in the morning at the same time with all the volunteers. The subjects were also asked to avoid eating or drinking anything different from water for at least 1 h before cell collection. Cells were obtained by scraping the internal face of both cheeks for 20 s each with a sterile cotton swab which was then transferred into 10 mL of TBS 0.05M (pH 7.6). Buccal cells from all volunteers were pooled and transferred into the buffer. TBS buffer solution was prepared with 6.05 g of Tris mixed with 8.76 g of NaCl in 1000 mL of deionized milliQ H_2_O (18 mΩ.cm^−1^). The pH was adjusted to 7.6 with 1 M HCl. TBS solution was stored at 4 °C. The buccal cell mix was vortexed for 30 s and ultra-sonicated three times for 10 s to avoid aggregates. Cell viability analysis and counting were carried out by using Trypan blue dye (0.4% solution) and a Neubauer chamber. The final cell concentration used was 5.104 cells/mL, and therefore 3.105 cells/vial. This amount was standardized for all the experiments and it was in the range described in previous works [46].

Both types of sampling procedures for collecting saliva and buccal cells were explained in detail to the subjects who also provided written consent to participate in the study. Previously, the Bioethical Committee of the Spanish National Council of Research (CSIC) approved this work. 

### 3.5. Artificial Saliva

Artificial saliva (AS) was prepared as previously shown [19]. To do so, a buffer solution was prepared by mixing the following components in 1000 mL of deionized milliQ H_2_O (18.2 mΩ.cm^−1^): NaHCO_3_ (5.208 g), K_2_HPO_4_.3H_2_O (1.369 g), NaCl (0.877 g), KCl (0.477 g), and CaCl_2_.2H_2_O (0.441 g) and 2.26 g of porcine mucin. AS was divided into 50 mL aliquots and frozen at −20 °C until further use. The pH of the mucin artificial saliva was adjusted to the pH of human saliva (7.2) by using NaOH and HCl.

### 3.6. Phenolic Compounds

Two phenolic compounds (catechin and gallic acid) were assayed. Two independent stock solutions of each compound were prepared (0.4 mg/mL) in hydroalcoholic solution (10% ethanol:water *v*/*v*) and tested at a final concentration in the headspace vial of 0.1 mg/mL, which had been already used in previous works [8,36].

Additionally, two commercial polyphenol extracts, one from grape seeds rich in procyanidins (Vitaflavan^®^) (D.R.T. Les Dèrives Resiniques and Terpéniques, Vielle-Saint-Girons, France) and the other one a red wine extract rich in anthocyanins (Provinols^®^) (Safic’Alcan Especialidades, S.A.U., Barcelona, Spain), were used. Both extracts had previously been used in different works and their chemical polyphenol composition fully characterized [39,40]. Previous to their use, an amount of each extract was placed in a glass vessel and submitted to an N_2_ current for 30 min in order to remove, as much as possible, endogenous aromas. Two independent stock solutions of each extract (4 mg/mL) were prepared in a hydroalcoholic solution (10% ethanol:water *v*/*v*). In agreement with previous works [9,18], a final concentration in the headspace vial of 1 mg/mL of each extract was tested. Figure 1 shows the different structures of the phenolic compounds used in the wines. 

### 3.7. Headspace Solid Phase Microextraction (HS-SPME) Procedure

As previously indicated [17], the estimation of the average ratio of liquid food/saliva in the human mouth has been shown to be 5:1 *w*/*v*. Blends containing the synthetic wines (hydroalcoholic solution, 10% ethanol, pH 3.5) and each aroma compound were poured in 20 mL headspace vials (Supelco). One milliliter of each of the tested oral components (HS, AS, BC) was also added to the wines to have a final volume in headspace vial of 6 mL. For some experiments, phenolic compounds were also added into the synthetic wines prior to the oral components. In all cases, a final volume of 6 mL and a liquid: saliva ratio of 5:1 was followed. The pHs of all the synthetic wines were very close (3.5 ± 0.2). 

The headspace vials were immediately sealed with Polytetrafluoroethylene (PTFE) septa in magnetic caps (Análisis Vínicos S.A, Spain). In order to avoid any aroma competition for protein binding sites, we chose to analyze aroma compounds one by one. Equilibrium times were previously established at 40 min for guaiacol and 30 min for all the other aroma compounds. The extraction procedure was automatically performed using a CombiPal system (CTC Analytics AG, Zwingen, Switzerland) with a 50/30 µM DVB/CAR/PDMS fiber of 2 cm length (Supelco, PA, USA). Samples were pre-incubated at 35 °C for 30 or 40 min depending on the compound and extraction was performed in the headspace of each vial for 2 min at 35 °C. Desorption was performed in the injector of the GC system (Agilent 6890N) in splitless mode for 10 min at 200 °C for all the compounds, except for β-ionone that was at 250 °C. After injection, the fiber was cleaned for 10 min at 270 °C to avoid any memory effect. All analyses were performed in triplicate.

### 3.8. GC/MS Analyses

Agilent MSD ChemStation software was used to control the system. For separation a DB-wax polar capillary column (60 m × 0.25 mm i.d. × 0.50 µM film thickness) from Agilent (J and W Scientific Folsom, CA, USA) was used. Helium was the carrier gas at a flow rate of 1 mL/min. A different program was set up for each compound. For the guaiacol analysis, the oven temperature started at 100 °C, holding for 5 min, and then it increased at 20 °C/min to 220 °C and held for 1 min. In the case of β-ionone, the oven temperature was initially held at 100 °C for 1 min, then increased at 20 °C/min to 200 °C and held for 15 min. Finally, in the case of ethyl hexanoate and β-phenylethanol, the oven temperature started at 50 °C and was held for 2 min. Then, it increased at a rate of 20 °C/min to 220 °C where it was held for 1 min. Run times were 11.5 min for ethyl hexanoate and β-phenyl ethanol, 12 min for guaiacol, and 22 min for β-ionone.

In the MS system (Agilent 5973N) the temperatures of the transfer line, quadrupole, and ion source were 270 °C, 150 °C, and 230 °C, respectively. Electron impact mass spectra were recorded at an ionization voltage of 70 eV and an ionization current of 10 µA. Acquisitions were performed in scan mode (from 35 to 350 amu) and Selective Ion Mode SIM mode. The mass spectra were compared with those from NIST 2.0 database. Since no internal standard was used, absolute peak areas (APAs) were obtained.

### 3.9. Statistical Analyses

One-way ANOVA was applied in order to determine the effect of specific oral components and/or phenolic compounds on wine aroma volatility. A Tukey test was performed for mean comparisons considering a significance level of *p* < 0.05. XLSTAT software v. 19.01 was used for these statistical analyses.

## 4. Conclusions

The use of static headspace analysis at equilibrium to study the interaction of aroma compounds with different types of oral components (centrifuged human saliva, artificial saliva with mucin, and buccal cells) in wines showed their significant effect on the volatility of four typical wine aroma compounds presenting different physicochemical characteristics (guaiacol, β-phenyl ethanol, ethyl hexanoate, and β-ionone). The extent of this effect depended on the type of aroma but also on the type of oral component. All tested aroma compounds showed higher volatiles in the wine systems containing BC. However, large differences in aroma volatility were found between wines with HS or AS. The lower volatility of some compounds (guaiacol, ethyl hexanoate) found in wines with HS can be related to their metabolic transformation by salivary enzymes; contrarily, the retention of the hydrophobic β-ionone by mucin present in AS can explain its lower volatility in this type of wines. 

Phenolic compounds also showed a different effect on aroma volatility depending on the type of odorants and the presence of different oral components in the wine. In general, gallic acid had little effect on polar compounds but it enhanced the volatility of the most hydrophobic ones (ethyl hexanoate and β-ionone). Furthermore, flavonoids significantly reduced the volatility of both polar (guaiacol and β-phenyl ethanol) and hydrophobic compounds (β-ionone in HS and BC wines), but through different mechanisms. The establishment of π–π interactions, in the case of the polar compounds (guaiacol, β-phenyl ethanol), or the formation of hydrophobic interactions in the case of β-ionone, are the most plausible mechanisms. However, flavonoid type polyphenols, and especially grape seed and wine extracts (constituted by procyanidins and anthocyanins respectively), enhanced the volatility of ethyl hexanoate, which might be due to the inhibition exerted by these phenolic compounds on some salivary enzymes (e.g., carboxyl esterase) involved in the metabolism of this odorant molecule. 

Overall, these results compare for the first time the impact of different types of oral components on wine aroma volatility, and also show the large relevance of these oral physiological components in the establishment of aroma–polyphenol interactions. Although the conditions used in this study do not exactly represent the dynamic situation of wine consumption, these results are highly valuable to help us in the understanding of the retronasal aroma release behavior of wine odorants during wine tasting. 

## Figures and Tables

**Table 1:** Structure and physicochemical properties of the aroma compounds employed in this study.

Compound	Chemical Structure	CAS Number	MW ^(a)^ (g mol^−1^)	BP ^(b)^ (°C)	*Log P* ^(c)^	OT ^(d)^ (µg/L)	Descriptor ^(e)^
Guaiacol	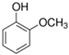	90-05-1	124	211	1.34	9.5–10	spice, clove
β-phenyl ethanol	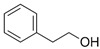	60-12-8	122	224	1.57	14,000–100,000	honey, spice, rose
Ethyl hexanoate	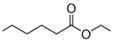	123-66-0	144	167	2.83	5–14	apple, peel, fruit
β-ionone	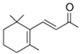	8013-90-9	192	262	4.42	0.09	raspberry, violet, flower

(a) Molecular weight, (b) boiling point, (c) *log P* (hydrophobicity): log of the octanol/water partition coefficient estimated from molecular modeling software EPI Suit (U.S EPA 2000–2007), (d) door thresholds compiled in Francis and Newton, 2008, (e) from Flavornet (http://www.flavornet.org) database, from NIST web chemistry book (2005) (http://www.webbook.nis.gov/chemistry).

## References

[1] Landy P., Druaux C., Voilley A. (1995). Retention of aroma compounds by proteins in aqueous solution. Food Chem..

[2] Pozo-Bayon M.A., Reineccius G. (2008). Interactions Between Wine Matrix Macro-Components and Aroma Compounds. Wine Chemistry and Biochemistry.

[3] Dufour C., Bayonove C.L. (1999). Influence of wine structurally different polysaccharides on the volatility of aroma substances in a model system. J. Agric. Food Chem..

[4] Dufour C., Bayonove C.L. (1999). Interactions between wine polyphenols and aroma substances. An insight at the molecular level. J. Agric. Food Chem..

[5] Dufour C., Sauvaitre I. (2000). Interactions between anthocyanins and aroma substances in a model system. Effect on the flavor of grape-derived beverages. J. Agric. Food Chem..

[6] Jung D.-M., De Ropp J.S., Ebeler S.E. (2000). Study of interactions between food phenolics and aromatic flavors using one- and two-dimensional (1)H NMR spectroscopy. J. Agric. Food Chem..

[7] Lorrain B., Tempere S., Iturmendi N., Moine V., De Revel G., Teissedre P.-L. (2013). Influence of phenolic compounds on the sensorial perception and volatility of red wine esters in model solution: An insight at the molecular level. Food Chem..

[8] Robinson A., E Ebeler S., Heymann H., Boss P.K., Solomon P., Trengove R. (2009). Interactions between Wine Volatile Compounds and Grape and Wine Matrix Components Influence Aroma Compound Headspace Partitioning. J. Agric. Food Chem..

[9] Villamor R.R., Evans M.A., Mattinson D.S., Ross C.F. (2013). Effects of ethanol, tannin and fructose on the headspace concentration and potential sensory significance of odorants in a model wine. Food Res. Int..

[10] Rodríguez-Bencomo J.J., González C.M., Andújar-Ortiz I., Martín-Álvarez P.J., Moreno-Arribas M.V., Pozo-Bayon M.A. (2011). Assessment of the effect of the non?volatile wine matrix on the volatility of typical wine aroma compounds by headspace solid phase microextraction/gas chromatography analysis. J. Sci. Food Agric..

[11] Saenz-Navajas M.-P., Campo E., Cullereé L., Zurbano P.F., Valentin D., Ferreira V. (2010). Effects of the Nonvolatile Matrix on the Aroma Perception of Wine. J. Agric. Food Chem..

[12] Pineau B., Barbe J.-C., Van Leeuwen K., Dubourdieu D. (2007). Which Impact for β-Damascenone on Red Wines Aroma?. J. Agric. Food Chem..

[13] Pozo-Bayon M.A., González C.M., Esteban-Fernández A. (2016). Wine Preference and Wine Aroma Perception. Wine Safety, Consumer Preference, and Human Health.

[14] Esteban-Fernández A., Rocha-Alcubilla N., González C.M., Moreno-Arribas M.V., Pozo-Bayon M.A. (2016). Intra-oral adsorption and release of aroma compounds following in-mouth wine exposure. Food Chem..

[15] Perez-Jiménez M., Chaya C., Pozo-Bayon M.A. (2019). Individual differences and effect of phenolic compounds in the immediate and prolonged in-mouth aroma release and retronasal aroma intensity during wine tasting. Food Chem..

[16] Muñoz-González C., Canon F., Feron G., Guichard E., Pozo-Bayon M.A. (2019). Assessment Wine Aroma Persistence by Using an in Vivo PTR-ToF-MS Approach and Its Relationship with Salivary Parameters. Molecules.

[17] Genovese A., Piombino P., Gambuti A., Moio L. (2009). Simulation of retronasal aroma of white and red wine in a model mouth system. Investigating the influence of saliva on volatile compound concentrations. Food Chem..

[18] Mitropoulou A., Hatzidimitriou E., Paraskevopoulou A. (2011). Aroma release of a model wine solution as influenced by the presence of non-volatile components. Effect of commercial tannin extracts, polysaccharides and artificial saliva. Food Res. Int..

[19] González C.M., Feron G., Guichard E., Rodríguez-Bencomo J.J., Martín-Álvarez P.J., Moreno-Arribas M.V., Pozo-Bayón M. (2014). Ángeles Understanding the Role of Saliva in Aroma Release from Wine by Using Static and Dynamic Headspace Conditions. J. Agric. Food Chem..

[20] Friel E.N., Taylor A.J. (2001). Effect of salivary components on volatile partitioning from solutions. J. Agric. Food Chem..

[21] Pagès-Hélary S., Andriot I., Guichard E., Canon F. (2014). Retention effect of human saliva on aroma release and respective contribution of salivary mucin and α-amylase. Food Res. Int..

[22] Ployon S., Morzel M., Canon F. (2017). The role of saliva in aroma release and perception. Food Chem..

[23] Buettner A. (2002). Influence of Human Salivary Enzymes on Odorant Concentration Changes Occurring in Vivo. 1. Esters and Thiols. J. Agric. Food Chem..

[24] Buettner A. (2002). Influence of Human Saliva on Odorant Concentrations. 2. Aldehydes, Alcohols, 3-Alkyl-2-methoxypyrazines, Methoxyphenols, and 3-Hydroxy-4,5-dimethyl-2(5H)-furanone. J. Agric. Food Chem..

[25] Ijichi C., Wakabayashi H., Sugiyama S., Ihara Y., Nogi Y., Nagashima A., Ihara S., Niimura Y., Shimizu Y., Kondo K. (2019). Metabolism of Odorant Molecules in Human Nasal/Oral Cavity Affects the Odorant Perception. Chem. Senses.

[26] González C.M., Brulé M., Feron G., Canon F. (2018). Does interindividual variability of saliva affect the release and metabolization of aroma compounds ex vivo? The particular case of elderly suffering or not from hyposalivation. J. Texture Stud..

[27] González C.M., Feron G., Brulé M., Canon F. (2017). Understanding the release and metabolism of aroma compounds using micro-volume saliva samples by ex vivo approaches. Food Chem..

[28] Pérez-Jiménez M., Rocha-Alcubilla N., Pozo-Bayon M.A. (2018). Effect of saliva esterase activity on ester solutions and possible consequences for thein-mouthester release during wine intake. J. Texture Stud..

[29] Canon F., Neiers F., Guichard E. (2018). Saliva and Flavor Perception: Perspectives. J. Agric. Food Chem..

[30] Isaac G., Koren E., Shalish M., Kanner J., Kohen R. (2012). Saliva increases the availability of lipophilic polyphenols as antioxidants and enhances their retention in the oral cavity. Arch. Oral Boil..

[31] Payne C., Bowyer P.K., Herderich M.J., Bastian S.E. (2009). Interaction of astringent grape seed procyanidins with oral epithelial cells. Food Chem..

[32] Jung D.-M., Ebeler S.E. (2003). Headspace Solid-Phase Microextraction Method for the Study of the Volatility of Selected Flavor Compounds. J. Agric. Food Chem..

[33] Aybeke E.N., Ployon S., Brulé M., De Fonseca B., Bourillot E., Morzel M., Lesniewska E., Canon F. (2019). Nanoscale Mapping of the Physical Surface Properties of Human Buccal Cells and Changes Induced by Saliva. Langmuir.

[34] Robert-Hazotte A., Schoumacker R., Semon E., Briand L., Guichard E., Le Quéré J.-L., Faure P., Heydel J.-M. (2019). Ex vivo real-time monitoring of volatile metabolites resulting from nasal odorant metabolism. Sci. Rep..

[35] Thiebaud N., Da Silva S.V., Jakob I., Sicard G., Chevalier J., Ménétrier F., Berdeaux O., Artur Y., Heydel J.-M., Le Bon A.-M. (2013). Odorant Metabolism Catalyzed by Olfactory Mucosal Enzymes Influences Peripheral Olfactory Responses in Rats. PLoS ONE.

[36] Hartmann P.J., McNair H.M., Zoecklein B.W. (2002). Measurement of 3-alkyl-2-methoxypyrazine by headspace solid-phase microextraction in spiked model wines. Am. J. Enol. Viticult..

[37] Gibbins H., Proctor G.B., Yakubov G., Wilson S., Carpenter G., Yakubov G. (2013). Concentration of salivary protective proteins within the bound oral mucosal pellicle. Oral Dis..

[38] Esteban-Fernández A., González C.M., Jiménez-Girón A., Pérez-Jiménez M., Pozo-Bayon M.A. (2017). Aroma release in the oral cavity after wine intake is influenced by wine matrix composition. Food Chem..

[39] Cueva C., Sánchez-Patán F., Monagas M., Walton G.E., Gibson G.R., Martín-Álvarez P.J., Bartolomé B., Moreno-Arribas M.V. (2012). In vitrofermentation of grape seed flavan-3-ol fractions by human faecal microbiota: Changes in microbial groups and phenolic metabolites. FEMS Microbiol. Ecol..

[40] Sánchez-Patán F., Cueva C., Monagas M., Walton G.E., Gibson M G.R., Quintanilla-López J.E., Lebrón-Aguilar R., Martín-Álvarez P.J., Moreno-Arribas M.V., Bartolomé B. (2012). In Vitro Fermentation of a Red Wine Extract by Human Gut Microbiota: Changes in Microbial Groups and Formation of Phenolic Metabolites. J. Agric. Food Chem..

[41] Hara Y., Honda M. (1990). The Inhibition of ?-Amylase by Tea Polyphenols. Agric. Boil. Chem..

[42] Kandra L., Gyémánt G., Zajácz Á., Batta G. (2004). Inhibitory effects of tannin on human salivary α-amylase. Biochem. Biophys. Res. Commun..

[43] Wang D., Zou L., Jin Q., Hou J., Ge G.-B., Yang L. (2018). Human carboxylesterases: A comprehensive review. Acta Pharm. Sin. B.

[44] Weng Z.-M., Ge G.-B., Dou T.-Y., Wang P., Liu P.-K., Tian X.-H., Qiao N., Yü Y., Zou L.-W., Zhou Q. (2018). Characterization and structure-activity relationship studies of flavonoids as inhibitors against human carboxylesterase 2. Bioorganic Chem..

[45] Criado C., Chaya C., Fernández-Ruíz V., Alvarez M.D., Herranz B., Pozo-Bayon M.A. (2019). Effect of saliva composition and flow on inter-individual differences in the temporal perception of retronasal aroma during wine tasting. Food Res. Int..

[46] Patel D., Smith A., Grist N., Barnett P., Smart J.D. (1999). An in vitro mucosal model predictive of bioadhesive agents in the oral cavity. J. Control. Release.

